# On the health paradox of occupational and leisure-time physical activity using objective measurements: Effects on autonomic imbalance

**DOI:** 10.1371/journal.pone.0177042

**Published:** 2017-05-04

**Authors:** David M. Hallman, Marie Birk Jørgensen, Andreas Holtermann

**Affiliations:** 1 Centre for Musculoskeletal Research, Department of Occupational and Public Health Sciences, University of Gävle, Gävle, Sweden; 2 National Research Centre for the Working Environment, Copenhagen, Denmark; Temple University, UNITED STATES

## Abstract

**Objective:**

Leisure-time physical activity (LTPA) has considerable benefits for cardiovascular health and longevity, while occupational physical activity (OPA) is associated with an elevated cardiovascular risk. This “health paradox” may be explained by different effects on the autonomic nervous system from OPA and LTPA. Thus, we aimed to investigate whether objectively measured OPA and LTPA are differentially associated with autonomic regulation among workers.

**Methods:**

The study comprised 514 blue-collar workers from the Danish cohort DPHACTO. Physical activity (i.e. walking, climbing stairs, running and cycling) was assessed objectively using accelerometers worn on the thigh, hip and trunk over multiple working days. During this period, a heart rate monitor was used to sample heart period intervals from the ECG signal. Heart rate and heart rate variability (HRV) indices were analyzed during nocturnal sleep as markers of autonomic regulation. Multiple regression analysis was used to determine the main effects of OPA and LTPA and their interaction on heart rate and HRV, adjusting for multiple confounders.

**Results:**

Statistically significant interaction was found between OPA and LTPA on heart rate (adjusted *p*<0.0001) and HRV indices in time (rMSSD, adjusted *p* = 0.004) and frequency-domains (HF, adjusted *p* = 0.022; LF, adjusted *p* = 0.033). The beneficial effect of LTPA on nocturnal heart rate and HRV clearly diminished with higher levels of OPA, and high levels of both OPA and LTPA had a detrimental effect.

**Conclusion:**

We found contrasting associations for objectively measured OPA and LTPA with heart rate and HRV during sleep. Differential effects of OPA and LTPA on autonomic regulation may contribute to the physical activity health paradox.

## Introduction

Physical activity has evident effects on cardiovascular health [1–3]. However, emerging research demonstrates that this effect differs depending on the domain of physical activity [4]. Leisure-time physical activity (LTPA) reduces the risk for cardiovascular diseases (CVD) and mortality [5, 6], while high occupational physical activity (OPA) is associated with risk elevation [7–11].

Because the mechanism behind the contrasting effects of OPA and LTPA on CVD and mortality risk is unknown, it is termed “the physical activity health paradox” [12]. A plausible hypothesis is that the different characteristics of OPA and LTPA impose dissimilar effects on the autonomic nervous system, which is closely involved in cardiovascular regulation, adaptation and health. LTPA is characterized by discretionary activities with sufficient time for rest and recovery, well known to improve autonomic regulation [13–15]. On the contrary, OPA is characterized by constrained activities related to the work tasks and productivity, with little opportunity for the worker to rest at convenience. Such physical activity with insufficient recovery may impose an elevated allostatic load [16] causing imbalanced autonomic regulation between the sympathetic and parasympathetic drives [17]. Imbalanced autonomic regulation is well documented to be associated with an elevated CVD risk [18]; presumably via different mediators, such as reduced baroreceptor activity, endothelial dysfunction [19], elevated blood pressure [20], and excess pro-inflammatory cytokines [21, 22].

Resting heart rate is a rough indicator of autonomic cardiac activity, and is a significant predictor for cardiovascular mortality [23, 24], particularly when derived during nocturnal sleep [25]. Heart rate variability (HRV) indices derived from ECG records during controlled rest or during nocturnal sleep are established indicators of parasympathetic (vagal) and sympathetic cardiac modulations. Diminished HRV indicating autonomic imbalance predicts CVD and all-cause mortality both in the general and clinical populations [26–31]. Thus, analyses of HRV indices combined with heart rate during nocturnal sleep may provide further insight into the effects of OPA and LTPA on intrinsic cardiovascular autonomic regulation.

Previous studies on the relationship between OPA and LTPA with cardiovascular health are limited to self-reported measurements of physical activity [32]. Objective measurements of physical activity are crucial for obtaining precise exposure estimates and minimize potential bias associated with self-reported measures of physical activity [33]. Moreover, because OPA and LTPA are correlated with socio-economic position, effect modification and confounding by socio-economic factors are an issue in the investigation of LTPA, OPA and health. The health effects of LTPA and OPA are therefore recommended to be conducted in homogeneous study populations with respect to socioeconomic position, like among blue-collar workers only [32].

The aim of the present study was to investigate if objectively measured OPA and LTPA are differentially associated with cardiac autonomic modulation during sleep among blue-collar workers. We hypothesized that OPA and LTPA would be inversely associated with autonomic regulation.

## Materials and methods

### Design and study population

The protocol of the Danish PHysical ACTivity cohort with Objective measurements (DPHACTO) was previously described [34]. Data was collected from April 2012 to May 2014. Objective data on physical activity and HRV were collected over multiple working days from a cross-sectional sample of Danish blue-collar workers representing three occupational sectors (cleaning, manufacturing and transportation). The workplaces were selected specifically to obtain a homogenous working population with respect to socioeconomic status, yet with a sufficient dispersion in physical activity at work. Workplaces were included if the organization allowed measurements during paid working hours. The inclusion criterion for the participants was to report blue-collar work as their current main occupation. Exclusion criteria were reporting predominant white-collar work, managing position, pregnancy, or allergy to adhesives.

In total, 2107 employees from 15 companies were invited to participate. A total of 901 blue-collar workers were considered eligible of whom 755 participated in the objective measurements of physical activity (accelerometry), resulting in valid measures (explained below) from 662 workers. Of the 622, 521 wore a heart rate monitor, resulting in acceptable HRV records in 514 workers. Thus, the analyzed population comprised 514 workers.

All participants provided their written informed consent prior to participation. The present study was conducted according to the Helsinki Declaration, approved by the Danish data protection agency, and evaluated by the Regional Scientific Ethics Committee of Copenhagen, Denmark (H-2-2012-011).

### Procedure

The data collection is described elsewhere in more detail [34, 35]. At baseline, participants filled out a brief questionnaire, underwent a health check and a physical examination, and participated in objective diurnal measurements of physical activity and heart rate. The participants were asked to wear four accelerometers and a heart rate monitor around the clock during four to five days, including at least two working days. The participants were instructed to wear the equipment the whole measurement period, and to perform a reference measurement in upright standing position for 15 s each day, to optimize activity detection from the accelerometer signals. They were also instructed to remove the equipment if it caused any kind of discomfort. During the measurement, the participant used a written diary to note working hours, leisure-time, and sleep, as well as time of the reference measurements. After the data collection, the equipment was returned to the research staff.

### Objective assessment of physical activity

The participants wore tri-axial accelerometers (Actigraph GT3X+, ActiGraph LLC, Florida, USA) attached on the thigh, dominant upper arm, hip, and trunk. The devices, attachment, and the processing and analysis of the accelerometer signals are described in detail elsewhere [36, 37]. The accelerometers were initialized using the Actilife software version 5.5 (ActiGraph LLC, Pensacola, FL, USA). Physical activity data were processed off-line using the Acti4 software (The National Research Centre for the Working Environment, Copenhagen, Denmark and BAuA, Berlin, Germany). This software classifies different physical activities and body postures with a high sensitivity and specificity, both in standardized and free-living conditions [37–40].

Non-wear was identified when (a) the software detected a period longer than 90 minutes with zero acceleration counts, or (b) the participant reported non-wear time, or (c) artefacts or missing data were detected by visual inspection. Non-work days and time in bed were excluded from further analyses. Valid work and leisure time intervals (determined from the diary) had to contain at least four h/day of accelerometer wear-time or 75% of the average wear-time across days for the individual. Recordings were excluded if they contained less than one complete day [36].

Different types of physical activity (e.g. lying, sitting, standing still, fast and slow walking, climbing stairs, running and cycling) were detected from the processed accelerometer signals, as previously described [38, 39]. The total time (h/day) spent in walking, climbing stairs, running, and cycling was summed up, and expressed in percentage of total time at work (OPA) and leisure (LTPA), respectively. The relative measures of OPA and LTPA were used in further statistical analyses to minimize the effect of wear time on physical activity. In addition, percent time in OPA and LTPA were divided into tertiles of low, middle and high exposure as a means to facilitate interpretation of the interaction between continuous measures of OPA and LTPA. The mean exposure for the low, middle and high tertiles was 11%, 19% and 27% for OPA, and 6%, 10% and 15% for LTPA, respectively (see S1 File for information on boundaries).

### Assessment of heart rate variability during sleep

The Actiheart monitor (CamNtech Ltd, UK) was used to collect interbeat ‘R‒R’ intervals (RRI) from the electrocardiogram (ECG) based on a standard two-led configuration attached using pre gelled electrodes [41]. The Actiheart is a small, water resistant device designed for long-term recordings over multiple days. R-peaks were detected online at 128Hz, and RRI values were then obtained with a resolution of 1 ms using an interpolation algorithm [42]. Beats for which the RRI deviated by more than 15% from adjacent normal beats were classified as abnormal and removed.

The analysis and data processing of HRV was done according to previously described procedures [41]. HRV indices in both the time and frequency domains were derived from normal-to-normal RRIs using non-overlapping 5-minute windows, according to the Task Force of the European Society of Cardiology and the North American Society of Pacing and Electrophysiology [43]. The time domain HRV indices calculated were: the root mean squared successive differences of RRI (RMSSD) and the standard deviation of RRI (SDNN). The frequency domain HRV indices calculated were: low frequency power (LF, 0.04–0.15 Hz), high frequency power (HF, 0.15–0.4 Hz) and normalized LF: $LFnu=\frac{LF}{\left(LF+HF\right)}$. A robust period detection procedure (RPD) was used to obtain the HRV power spectral components [44, 45].

Based on the diary and accelerometer data, HRV was analyzed during periods classified as nocturnal sleep without movement [41]. The three 5-minute intervals with the lowest RRI were detected for each sleep interval, and their mean heart rate and HRV values were determined and averaged across days. Non-normal distributions were found for RMSSD, LF, and HF which were transformed using the natural logarithm (ln).

### Assessment of potential confounders

Potential confounders were selected a priori based on theoretical and empirical indications of their relationship with physical activity and autonomic regulation. The following variables were assessed based on the questionnaire: age (in years, based on civil registration number); gender (“male” or “female”); smoking using a single item with four categories dichotomized into “yes” (daily smoking) or “no” (occasionally, formerly or never smoked); social support at work based on the COPSOQ short-form [46], using two items (5-point scale) which were summed up, with higher values reflecting better social support; seniority in the current job (years); current use of cardiovascular drugs (“yes” or “no”) using two items about anti-hypertensives and heart/lung disease medication. The following variables were obtained using objective measurements: body mass index (BMI, kg·m^-2^) calculated from objectively measured height (cm) and body weight (kg); resting systolic and diastolic blood pressure (mmHg) obtained from three readings using the Omron M6 Comfort (Omron health Care, Netherlands) on the upper left arm after 15 min of seated rest; percent time in sitting and standing during work and leisure, based on the accelerometer recordings [47].

### Statistical analyses

SPSS version 22 (IBM) was used for the statistical analyses. Descriptive data are presented as mean and standard deviation (SD) between subjects or as frequencies and percent were appropriate.

Multiple linear regression analysis was used to determine the association between physical activity (percentages of OPA and LTPA) and heart rate and HRV indices during sleep. Prior to the analysis, OPA and LTPA were centered by subtracting the population mean value from each subject to avoid multicollinearity. The crude model (model 1) contained the independent variables OPA, LTPA and their multiplicative interaction (OPA × LTPA). Dependent variables were heart rate, RMSSD (ln), SDNN, LF (ln), HF (ln) and LFnu, which were included in separate regression models. In a second adjusted model (model 2), the initial models were re-run with adjustments for age, gender, BMI and current smoking.

A sensitivity analysis was performed to further examine the multiplicative interaction between OPA and LTPA in stratified regression models based on tertiles (low, middle and high) of OPA against heart rate and HRV indices using continuous LTPA as a predictor. Also, the same stratified models were constructed for tertiles of LTPA using continuous OPA as a predictor. Further, to test the robustness of the primary adjusted results, model 2 was constructed with additional adjustment for sleep duration and social support. To examine possible effect modification by psychosocial factors, model 2 was also performed with stratification for social support at work (median split).

In additional sensitivity analyses, the primary adjusted model (model 2) was re-run with stratification by CVD, i.e. reporting current use of cardiovascular disease medication and/or having hypertension (≥140/90 mmHg). Also, stratified models were performed among workers with controlled (using antihypertensive medication) and uncontrolled hypertension.

Inspection of the residuals from each regression model indicated no deviation from normality, and there was no sign of multicollinearity among the predictors. For each regression model we derived the estimate (B), standard error (SE), and *p*-value. The level of significance was set at *p*<0.05.

## Results

Descriptive information on the Dphacto study population is presented in Table 1. The study population of 514 blue-collar workers comprised three occupational sectors (cleaning, manufacturing and transportation) with different distributions of males and females. The population was of middle age, slightly overweight and had worked in their current job close to 15 years on average. Eighteen percent reported using medication for hypertension and/or heart/lung disease, and 19% were hypertensive (≥140/90 mmHg). Among those with hypertension, only 26% used antihypertensive medication. In total, 32% (n = 155) of the workers were on CVD medication and/or were hypertensive. Percent time in physical activity was on average 19% and 10% for OPA and LTPA, respectively. Recommended levels (≥30 minutes/day) of moderate-to-vigorous physical activity were achieved by 91% at work and 71% during leisure. Detailed information of OPA and LTPA, including specific activity types, postures and intensities, are presented in the Supporting file (S1 File). Resting heart rate derived during nocturnal sleep was on average 57 (SD 7) bpm. The HRV indices are presented in Table 2.

**Table 1:** Descriptive data of the analyzed study population in Dphacto.

	N	n	%	Mean	SD
**Gender, female**	514	224	44		
**Sector**	514				
**Cleaning**		105	20		
**Manufacturing**		367	72		
**Transportation**		42	8		
**Current smoking**	502	123	24		
**Age (years)**	514			45.3	9.8
**BMI (kg·m**^**-2**^**)**	502			27.4	4.8
**Job seniority (years)**	493			13.7	10.5
**Social support at work (scale 0–8)**	346			6.2	1.3
**Physical activity**^**a**^	514				
**OPA (% of work hours)**				18.9	7.2
**LTPA (% of leisure-time)**				10.4	4.2
**OPA (h/day)**				1.4	0.6
**LTPA (h/day)**				0.9	0.4
**Achieving recommended OPA levels**^**b**^	514	469	91		
**Achieving recommended LTPA levels**^**b**^	514	364	71		
**Measurement times**	514				
**Work (h/day)**				7.6	1.2
**Leisure (h/day)**				8.8	1.5
**Sleep (h/day)**				7.1	1.0
**Wear-time accelerometer (days)**				2.7	1.0
**Wear-time heart rate monitor (days)**				2.4	0.9
**Medication**	514				
**Antihypertensives**^**c**^		70	14		
**Heart and lung disease medication**^**c**^		36	7		
**Antidepressants**^**d**^		15	3		
**Analgesics**^**d**^		98	19		
**Other**^**d**^		118	23		
**Systolic blood pressure (mmHg)**	513			133.7	14.7
**Diastolic blood pressure (mmHg)**	513			83.9	10.9
**Hypertension (>140/90 mmHg)**	513	95	19		
**Uncontrolled hypertension**	513	70	14		

Abbreviations: BMI, body mass index; OPA, occupational physical activity; LTPA, leisure-time physical activity.

^a^Physical activity (i.e. walking, running, cycling and climbing stairs) is expressed as percentage of accelerometer wear-time during work and leisure.

^b^Accumulating ≥ 30 minutes per day of moderate-to-vigorous physical activity (i.e. fast walking, running, cycling and climbing stairs).

^c^Currently on medication.

^d^During the past 3 months.

**Table 2:** Mean and standard deviation (SD) of heart rate and heart rate variability (HRV) indices during nocturnal sleep in the Dphacto study population.

	n	Mean	SD
**Heart rate (bpm)**	514	56.7	7.5
**RMSSD (ms)**	514	50.6	28.9
**ln RMSSD (ln ms)**	514	3.8	0.6
**SDNN (ms)**	514	55.9	22.5
**LF (ms**^**2**^**)**	514	962.0	1077.6
**ln LF (ln ms**^**2**^**)**	514	6.5	0.9
**HF (ms**^**2**^**)**	514	1074.4	1430.8
**ln HF (ln ms**^**2**^**)**	514	6.3	1.2
**LFnu**	514	0.5	0.2

Abbreviations: RMSSD, root mean squared successive differences between RR intervals; SDNN, standard deviation of RR intervals; LF, low frequency power, HF, high frequency power; LFnu, LF in normalized units.

### Association between physical activity and heart rate during sleep

**Fig 1:**
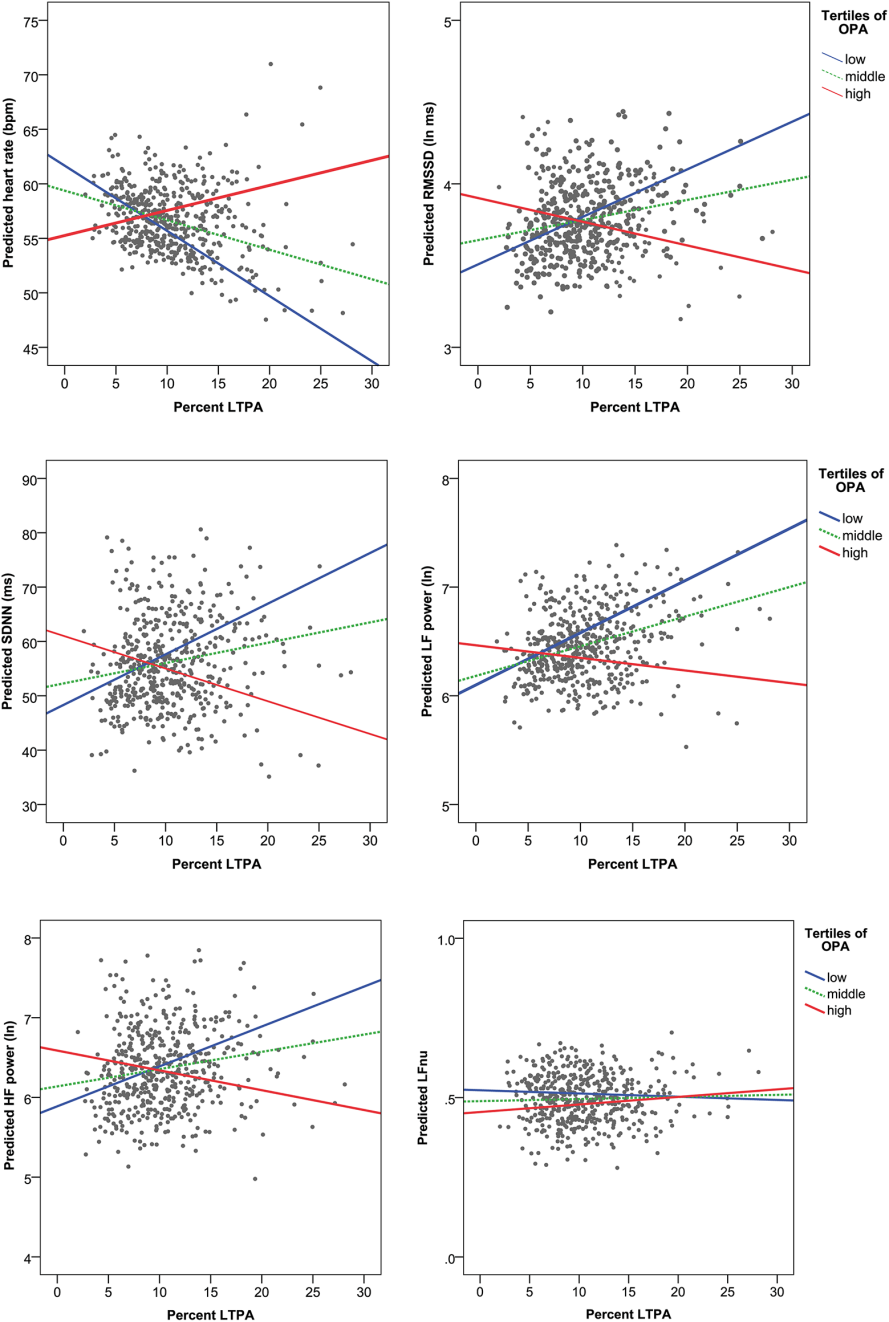
Predicted nocturnal heart rate and heart rate variability indices from the multiple regression analyses adjusted for age, gender, body-mass index and smoking. The x-axes represent percent time in leisure-time physical activity (LTPA), and the lines represent tertiles (low, middle and high) of occupational physical activity (OPA).

We found a statistically significant interaction effect between percent time spent in OPA and LTPA on heart rate during sleep (*p*<0.0001), which persisted with adjustments for potential confounders (Table 3). This interaction effect is illustrated in Fig 1, where the linear association between LTPA and heart rate is seen to clearly depend on the level of OPA. Also, the stratified models (S1 and S2 Tables) indicated a significant negative association between LTPA and heart rate only for the low tertile of OPA (adjusted B = -5.99, *p*<0.0001), while a positive association between OPA and heart rate was present for the middle (B = 1.50, p = 0.088) and high tertile of LTPA (adjusted B = 2.48, *p* = 0.001).

**Table 3:** Multiple linear regression with crude (model 1) and adjusted (model 2) associations for occupational (OPA) and leisure-time physical activity (LTPA) with heart rate and heart rate variability indices during sleep.

	Model 1 (n = 514)			Model 2 (n = 488)		
	B	SE	*p*	B	SE	*p*
**Heart rate (bpm)**						
**OPA**	1.10	0.45	0.016	0.87	0.45	0.054
**LTPA**	-2.17	0.77	0.005	-1.77	0.77	0.022
**Interaction**	0.51	0.10	<0.0001	0.49	0.10	<0.0001
**RMSSD (ln ms)**						
**OPA**	-0.03	0.03	0.376	-0.04	0.03	0.235
**LTPA**	0.10	0.06	0.079	0.05	0.06	0.379
**Interaction**	-0.02	0.01	0.007	-0.02	0.01	0.004
**SDNN (ms)**						
**OPA**	-1.98	1.40	0.156	-1.81	1.37	0.189
**LTPA**	2.59	2.37	0.275	0.56	2.35	0.811
**Interaction**	-0.80	0.32	0.012	-0.74	0.31	0.019
**LF (ln ms**^**2**^**)**						
**OPA**	-0.16	0.06	0.004	-0.13	0.06	0.017
**LTPA**	0.23	0.10	0.014	0.16	0.10	0.087
**Interaction**	-0.03	0.01	0.016	-0.03	0.01	0.033
**HF (ln ms**^**2**^**)**						
**OPA**	-0.06	0.07	0.436	-0.08	0.07	0.225
**LTPA**	0.18	0.12	0.137	0.07	0.12	0.526
**Interaction**	-0.04	0.02	0.033	-0.04	0.02	0.022
**LFnu**						
**OPA**	-0.02	0.01	0.067	-0.01	0.01	0.430
**LTPA**	0.00	0.02	0.816	0.01	0.02	0.488
**Interaction**	0.00	0.00	0.747	0.00	0.00	0.437

Note: Estimates (B) represent change in HRV indices with 10 unit increments in percent time in OPA and LTPA, which were centered prior to the analysis; Interaction represents OPA × LTPA; Model 1: unadjusted model. Model 2: adjusted for age, gender, body-mass index and current smoking. Abbreviations: RMSSD, root mean squared successive differences between RR intervals; SDNN, standard deviation of RR intervals; LF, low frequency power, HF, high frequency power; LFnu, LF in normalized units.

Accordingly, the main effects of OPA (1 bpm, *p* = 0.016) and LTPA (-2 bpm, *p* = 0.005) on heart rate were statistically significant in the crude model (Table 3, model 1) revealing a positive association with heart rate for OPA and a negative association for LTPA, with slightly reduced estimates in the adjusted model.

### Association between physical activity and heart rate variability during sleep

We found a statistically significant interaction effect between OPA and LTPA for all HRV indices during sleep (all *p*<0.05) apart from LFnu (*p* = 0.75), both in the crude and adjusted models (Table 3, models 1–2). As illustrated in Fig 1, the linear association between LTPA and HRV clearly depended on the level of OPA. The stratified models (S1 and S2 Tables) indicated significant positive associations between LTPA and HRV indices for the low (RMSSD and HF: adjusted *p*<0.05) and middle (LF: adjusted *p* = 0.032) tertiles of OPA, while non-significant negative estimates were found for high OPA. Inversely, significant negative associations between OPA and HRV were present for the middle (SDNN and LF: adjusted *p*<0.05) and high (RMSSD, SDNN, LF and HF: adjusted *p*<0.05) tertiles of LTPA, while nonsignificant positive estimates were found for low LTPA.

Significant and opposite main effects of OPA and LTPA on HRV were found for LF power only (OPA *p* = 0.004; LTPA *p* = 0.014). That is, OPA was negatively associated with LF, while LTPA showed a positive association (Table 3, model 1). This main effect remained significant in the adjusted model for OPA, and was border-line significant for LTPA (model 2).

### Sensitivity analyses

**Fig 2:**
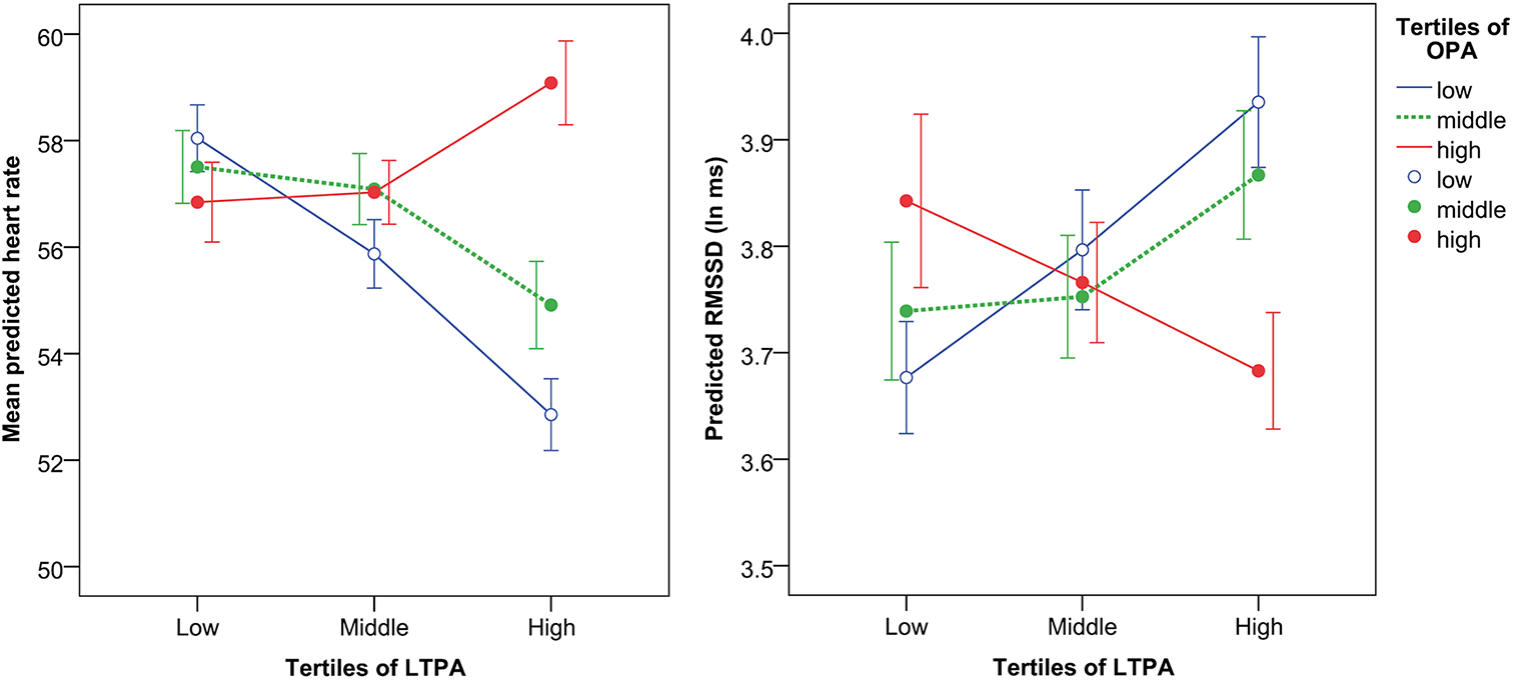
Mean predicted heart rate and RMSSD (parasympathetic index) across tertiles (low, middle and high) of occupational (OPA) and leisure-time physical activity (LTPA). Lines represent tertiles of OPA, and the y-axis represents tertiles of LTPA. Error bars represent 95% confidence intervals.

The association between continuous LTPA with heart rate and HRV indices was also modeled separately in each tertile (low, middle and high) of OPA. These results were consistent with the primary models indicating a beneficial association for LTPA with heart rate and HRV indices among those with low or middle OPA, while opposite estimates were found in the high OPA group (Fig 2, S1 Table). Similarly, the association between continuous OPA and heart rate and HRV was modeled across tertiles of LTPA. These results revealed unfavorable associations for OPA with heart rate (positive estimate) and HRV indices (negative estimates) among those with middle and high LTPA, while the opposite associations were found in the low tertile of OPA (S2 Table).

To further test the consistency of the primary adjusted models, we performed additional adjustments for sleep duration and psychosocial factors (social support at work), and it did not change the estimates or significance of the interaction between OPA and LTPA to any marked extent (all *p*<0.05; LFnu *p*>0.05). To account for possible effect modification by psychosocial factors, the same models were performed with stratification by social support at work, revealing consistent estimates for the interaction among workers with low and high levels of support (S3 Table).

To test if the interaction between OPA and LTPA on heart rate and HRV was consistent when accounting for the occurrence of cardiovascular ailments, the primary adjusted models were re-run with stratification by the occurrence of CVD, i.e. hypertension (≥140/90 mmHg) and/or cardiovascular disease medication (n = 155). OPA and LTPA showed stronger interactions among persons with CVD for most outcomes (heart rate, RMSSD, HF and LFnu) than among persons without (S4 Table). Also, comparable effect estimates were observed among those with controlled and uncontrolled hypertension, apart from LFnu showing positive estimates only for uncontrolled hypertension (S5 Table).

## Discussion

As far as we know, this is the first study investigating the independent and interactive associations for objectively measured OPA and LTPA with cardiac autonomic modulation during sleep (assessed by heart rate and HRV). We found in blue-collar workers that OPA and LTPA were inversely associated with autonomic regulation during sleep, and that the beneficial effect of LTPA on autonomic regulation was diminished with higher levels of OPA.

In agreement with previous studies [48], more LTPA was associated with reduced heart rate during sleep, as indicated by the significant main effect of LTPA in both the crude and adjusted models (Table 2). In contrast, higher levels of OPA were significantly associated with elevated heart rate, although the effect became marginally insignificant after adjustment for potential confounders (Table 2). Elevated heart rate during sleep is a strong predictor for CVD [25], and may therefore contribute to the increased cardiovascular risk from high OPA observed in previous studies [7, 10, 11]. The observed interaction between OPA and LTPA on heart rate during sleep (Table 2) indicates that the association between OPA, LTPA and heart rate depends on the activity level in the opposite domain. For instance, the effect of LTPA on heart rate diminished with increasing OPA (Figs 1 and 2). Thus, this result suggests that high LTPA may be beneficial for cardiovascular health among blue-collar workers only when OPA is low, while it may be even detrimental when combined with medium or high levels of OPA.

The HRV analysis revealed a positive association between LTPA and LF power suggesting enhanced sympathetic-baroreceptor cardiac modulation during sleep among workers with higher levels of LTPA [49]. In contrast, more OPA was significantly associated with reduced LF power, which indicates that high OPA is associated with attenuated cardiac autonomic modulation during sleep, although this association became less significant after adjustment for potential confounders. In contrast to previous studies documenting a beneficial effect of physical activity on parasympathetic (vagal) activity [13–15], we could not find any significant association between physical activity and parasympathetic HRV indices (i.e. RMSSD and HF) or global HRV (i.e. SDNN). The interaction between OPA and LTPA was significant for all HRV indices, apart from LFnu, which remained after adjustments for a wide range of potential confounders (Table 2). This interaction indicates that the effect of LTPA on improved cardiac autonomic modulation (i.e. increased nocturnal HRV) during sleep gets weaker and even reversed with higher levels of OPA. Thus, performing high levels of physical activity both during work and leisure among blue-collar workers may result in autonomic imbalance, regardless of age, gender, body-mass index, smoking habits, and cardiovascular ailments. Autonomic imbalance as indicated by elevated heart rate and diminished HRV may impose an increased cardiovascular disease risk [18].

The apparently contrasting effects of OPA and LTPA on nocturnal autonomic activity among blue-collar workers may be explained by the different activity patterns during work and leisure in this occupational group [50]. Also, in blue-collar occupations comprising manual work tasks, OPA is generally more constrained and provides fewer opportunities for rest and recovery compared with LTPA. Thus, high amounts of OPA may impose a cardiovascular strain due to insufficient recovery [51–53], particularly when combined with high LTPA.

The use of objective technical measurements of physical activity is a clear strength of the current study as it allows precise exposure estimates while minimizing potential bias associated with self-reported measures of physical activity [33]. Since the use of activity classification from multiple accelerometers is a rather novel approach in observational studies, comparisons with current recommended guidelines for time and intensity of physical activity are limited. Still, we found that even though the workers in the highest tertile of OPA and LTPA achieved recommended levels (i.e. ≥30 minutes per day) of moderate-to-vigorous physical activity (Table 2 and S1 File), the cardiovascular benefits differed considerably depending on the activity level in the opposite domain.

Our definition of physical activity comprised common dynamic activities, such as walking, running and cycling. However, we did not evaluate the effect of other specific activities of presumed importance to cardiovascular health, such as sitting and standing, which must be acknowledged as a limitation of the study.

Another limitation of the study is the cross-sectional design precluding causal inferences. Still, to partly account for reversed causality, we performed a sensitivity analysis stratified by cardiovascular disease medication and hypertension, and found consistent results. Thus, it appears likely that the physical activity affected cardiovascular health rather than the other way around.

To rule out the acute effects of physical activity on autonomic activity, heart rate and HRV indices were derived during nocturnal sleep. Still, we could not completely separate short and long-term effects of physical activity on nocturnal autonomic regulation. Thus, excessive physical activity and exercise during the day might have affected HRV during the following night [54].

We accounted for a range of potential confounders, including individual, occupational and life-style factors and cardiovascular health, which did not appear to influence the results to any marked extent. Still, residual confounding by non-measured factors cannot be completely ruled out. We did not measure respiration, which is known to influence HRV [49]. The respiration rate during sleep may be reduced close to 0.15 Hz, and the high frequency parasympathetic cardiac modulations may shift toward the LF spectral component. Thus, the frequency domain indices of HRV should be interpreted with caution. Further, although social support at work did not influence the results, we lacked measures of psychosocial factors during leisure time, which is a possible limitation.

### Conclusion

We found among blue-collar workers that objectively measured OPA and LTPA were inversely associated with cardiac autonomic regulation during sleep (indexed by heart rate and HRV), and that the beneficial effect of LTPA diminished among workers with higher levels of OPA. The contrasting and interactive effects of OPA and LTPA on cardiac autonomic modulation suggest that autonomic imbalance contributes to the health paradox of physical activity during work and leisure. Future prospective studies should investigate whether autonomic imbalance contributes to the contrasting relationship between OPA and LTPA with cardiovascular disease morbidity and mortality. Also, future studies on the health paradox of OPA and LTPA should address the role of specific activity types, including sitting and standing.

## Supporting information

S1 Table Association between percent time in leisure-time physical activity (LTPA) and heart rate variability indices during sleep stratified by low (n = 170), middle (n = 171) and high (n = 173) levels of occupational physical activity (OPA). Abbreviations: RMSSD, root mean squared successive differences between RR intervals; SDNN, standard deviation of RR intervals; LF, low frequency power, HF, high frequency power; LFnu, LF in normalized units.
Model 1: unadjusted model.
Model 2: adjusted for age, gender, body mass index and current smoking.
Estimates (B) represent change in HRV indices with 10 unit increments in percent LTPA.
(DOCX)

S2 Table Association between percent time in occupational physical activity (OPA) and heart rate variability indices during sleep stratified by low (n = 169), middle (n = 173) and high (n = 172) levels of leisure-time physical activity (LTPA). Abbreviations: RMSSD, root mean squared successive differences between RR intervals; SDNN, standard deviation of RR intervals; LF, low frequency power, HF, high frequency power; LFnu, LF in normalized units.
Model 1: unadjusted model.
Model 2: adjusted for age, gender, body mass index and current smoking.
Estimates (B) represent change in HRV indices with 10 unit increments in percent OPA.
(DOCX)

S3 Table Associations for occupational (OPA) and leisure-time physical activity (LTPA) with heart rate and heart rate variability indices during sleep, stratified by Social support at work. Note: Estimates (B) represent change in HRV indices with 10 unit increments in percent time in OPA and LTPA, which were centered prior to the analysis; Interaction represents OPA × LTPA; the models are adjusted for age, gender, body-mass index and current smoking.
Abbreviations: RMSSD, root mean squared successive differences between RR intervals; SDNN, standard deviation of RR intervals; LF, low frequency power, HF, high frequency power; LFnu, LF in normalized units.
(DOCX)

S4 Table Associations for occupational (OPA) and leisure-time physical activity (LTPA) with heart rate and heart rate variability indices during sleep, stratified by the occurrence of cardiovascular disease (CVD). Note: Estimates (B) represent change in HRV indices with 10 unit increments in percent time in OPA and LTPA, which were centered prior to the analysis; Interaction represents OPA × LTPA; the models are adjusted for age, gender, body-mass index and current smoking.
Abbreviations: RMSSD, root mean squared successive differences between RR intervals; SDNN, standard deviation of RR intervals; LF, low frequency power, HF, high frequency power; LFnu, LF in normalized units.
(DOCX)

S5 Table Associations for occupational (OPA) and leisure-time physical activity (LTPA) with heart rate and heart rate variability indices during sleep, stratified by controlled hypertension. Note: Estimates (B) represent change in HRV indices with 10 unit increments in percent time in OPA and LTPA, which were centered prior to the analysis; Interaction represents OPA × LTPA; the models are adjusted for age, gender, body-mass index and current smoking.
Abbreviations: RMSSD, root mean squared successive differences between RR intervals; SDNN, standard deviation of RR intervals; LF, low frequency power, HF, high frequency power; LFnu, LF in normalized units.
(DOCX)

S1 File Data set, boundaries of physical activity tertiles, descriptive data of physical activity types, meeting activity recommendations, and questionnaire. (XLSX)
