# Supplementary material for: On the health paradox of occupational and leisure-time physical activity using objective measurements: Effects on autonomic imbalance
Source: PLoS One. 2017 May 4;12(5):e0177042. doi: 10.1371/journal.pone.0177042 (PMC5417644; doi:10.1371/journal.pone.0177042)
Supplement: S1 Table — Abbreviations: RMSSD, root mean squared successive differences between RR intervals; SDNN, standard deviation of RR intervals; LF, low frequency power, HF, high frequency power; LFnu, LF in normalized units. Model 1: unadjusted model. Model 2: adjusted for age, gender, body mass index and current smoking. Estimates (B) represent change in HRV indices with 10 unit increments in percent LTPA. (DOCX) [file pone.0177042.s001.docx]

**S1 Table.** **Association between percent time in leisure-time physical activity (LTPA) and heart rate variability indices during sleep stratified by low (n=170), middle (n=171) and high (n=173) levels of occupational physical activity (OPA).**

|  | **Model 1 (unadjusted)** |  |  | **Model 2 (adjusted)** |  |  |
| --- | --- | --- | --- | --- | --- | --- |
|  | **B** | **SE** | ***p*** | **B** | **SE** | ***p*** |
| **Heart rate (bpm)** |  |  |  |  |  |  |
| **Low OPA** | -6.26 | 1.38 | <0.0001 | -5.99 | 1.37 | <0.0001 |
| **Middle OPA** | -2.38 | 1.11 | 0.033 | -1.70 | 1.12 | 0.133 |
| **High OPA** | 2.11 | 1.49 | 0.158 | 2.14 | 1.54 | 0.166 |
| **RMSSD (ln ms)** |  |  |  |  |  |  |
| **Low OPA** | 0.27 | 0.10 | 0.008 | 0.22 | 0.10 | 0.030 |
| **Middle OPA** | 0.11 | 0.10 | 0.269 | 0.05 | 0.10 | 0.635 |
| **High OPA** | -0.08 | 0.10 | 0.455 | -0.10 | 0.10 | 0.304 |
| **SDNN (ms)** |  |  |  |  |  |  |
| **Low OPA** | 7.06 | 3.93 | 0.074 | 4.76 | 4.00 | 0.236 |
| **Middle OPA** | 4.33 | 4.02 | 0.282 | 1.57 | 4.06 | 0.700 |
| **High OPA** | -4.46 | 4.30 | 0.301 | -4.21 | 4.22 | 0.320 |
| **LF (ln ms^2^)** |  |  |  |  |  |  |
| **Low OPA** | 0.29 | 0.16 | 0.074 | 0.20 | 0.17 | 0.233 |
| **Middle OPA** | 0.46 | 0.16 | 0.004 | 0.36 | 0.17 | 0.032 |
| **High OPA** | -0.13 | 0.17 | 0.457 | -0.08 | 0.17 | 0.636 |
| **HF (ln ms^2^)** |  |  |  |  |  |  |
| **Low OPA** | 0.51 | 0.21 | 0.018 | 0.41 | 0.21 | 0.049 |
| **Middle OPA** | 0.18 | 0.21 | 0.400 | 0.03 | 0.20 | 0.897 |
| **High OPA** | -0.13 | 0.21 | 0.525 | -0.18 | 0.21 | 0.390 |
| **LFnu** |  |  |  |  |  |  |
| **Low OPA** | -0.05 | 0.04 | 0.175 | -0.05 | 0.03 | 0.143 |
| **Middle OPA** | 0.05 | 0.03 | 0.134 | 0.06 | 0.03 | 0.054 |
| **High OPA** | 0.00 | 0.03 | 0.925 | 0.02 | 0.03 | 0.461 |

Abbreviations: RMSSD, root mean squared successive differences between RR intervalss; SDNN, standard deviation of RR intervals; LF, low frequency power, HF, high frequency power; LFnu, LF in normalized units.

Model 1: unadjusted model.

Model 2: adjusted for age, gender, body mass index and current smoking.

Estimates (B) represent change in HRV indices with 10 unit increments in percent LTPA.
