# Supplementary material for: On the health paradox of occupational and leisure-time physical activity using objective measurements: Effects on autonomic imbalance
Source: PLoS One. 2017 May 4;12(5):e0177042. doi: 10.1371/journal.pone.0177042 (PMC5417644; doi:10.1371/journal.pone.0177042)
Supplement: S2 Table — Abbreviations: RMSSD, root mean squared successive differences between RR intervals; SDNN, standard deviation of RR intervals; LF, low frequency power, HF, high frequency power; LFnu, LF in normalized units. Model 1: unadjusted model. Model 2: adjusted for age, gender, body mass index and current smoking. Estimates (B) represent change in HRV indices with 10 unit increments in percent OPA. (DOCX) [file pone.0177042.s002.docx]

**S2 Table.** **Association between percent time in occupational physical activity (OPA) and heart rate variability indices during sleep stratified by low (n=169), middle (n=173) and high (n=172) levels of leisure-time physical activity (LTPA).**

|  | **Model 1 (unadjusted)** |  |  | **Model 2 (adjusted)** |  |  |
| --- | --- | --- | --- | --- | --- | --- |
|  | **B** | **SE** | ***p*** | **B** | **SE** | ***p*** |
| **Heart rate (bpm)** |  |  |  |  |  |  |
| **Low LTPA** | -1.43 | 0.92 | 0.121 | -0.96 | 0.92 | 0.299 |
| **Middle LTPA** | 1.49 | 0.86 | 0.085 | 1.50 | 0.88 | 0.088 |
| **High LTPA** | 2.76 | 0.74 | 0.000 | 2.48 | 0.72 | 0.001 |
| **RMSSD (ln ms)** |  |  |  |  |  |  |
| **Low LTPA** | 0.11 | 0.07 | 0.116 | 0.09 | 0.07 | 0.205 |
| **Middle LTPA** | -0.08 | 0.07 | 0.228 | -0.10 | 0.07 | 0.145 |
| **High LTPA** | -0.12 | 0.05 | 0.020 | -0.13 | 0.05 | 0.013 |
| **SDNN (ms)** |  |  |  |  |  |  |
| **Low LTPA** | 5.07 | 2.78 | 0.070 | 3.73 | 2.64 | 0.160 |
| **Middle LTPA** | -5.39 | 2.82 | 0.057 | -5.95 | 2.86 | 0.039 |
| **High LTPA** | -4.98 | 2.02 | 0.015 | -4.53 | 2.04 | 0.028 |
| **LF (ln ms^2^)** |  |  |  |  |  |  |
| **Low LTPA** | 0.07 | 0.11 | 0.532 | 0.06 | 0.11 | 0.582 |
| **Middle LTPA** | -0.23 | 0.11 | 0.039 | -0.24 | 0.11 | 0.040 |
| **High LTPA** | -0.28 | 0.09 | 0.002 | -0.25 | 0.09 | 0.005 |
| **HF (ln ms^2^)** |  |  |  |  |  |  |
| **Low LTPA** | 0.23 | 0.15 | 0.133 | 0.19 | 0.14 | 0.187 |
| **Middle LTPA** | -0.18 | 0.14 | 0.203 | -0.21 | 0.13 | 0.115 |
| **High LTPA** | -0.22 | 0.11 | 0.041 | -0.24 | 0.10 | 0.020 |
| **LFnu** |  |  |  |  |  |  |
| **Low LTPA** | -0.03 | 0.02 | 0.197 | -0.02 | 0.02 | 0.271 |
| **Middle LTPA** | -0.02 | 0.02 | 0.480 | -0.01 | 0.02 | 0.691 |
| **High LTPA** | -0.01 | 0.02 | 0.548 | 0.00 | 0.02 | 0.889 |

Abbreviations: RMSSD, root mean squared successive differences between RR intervals; SDNN, standard deviation of RR intervals; LF, low frequency power; HF, high frequency power; LFnu, LF in normalized units.

Model 1: unadjusted model.

Model 2: adjusted for age, gender, body mass index and current smoking.

Estimates (B) represent change in HRV indices with 10 unit increments in percent OPA.
