# Supplementary material for: On the health paradox of occupational and leisure-time physical activity using objective measurements: Effects on autonomic imbalance
Source: PLoS One. 2017 May 4;12(5):e0177042. doi: 10.1371/journal.pone.0177042 (PMC5417644; doi:10.1371/journal.pone.0177042)
Supplement: S3 Table — Note: Estimates (B) represent change in HRV indices with 10 unit increments in percent time in OPA and LTPA, which were centered prior to the analysis; Interaction represents OPA × LTPA; the models are adjusted for age, gender, body-mass index and current smoking. Abbreviations: RMSSD, root mean squared successive differences between RR intervals; SDNN, standard deviation of RR intervals; LF, low frequency power, HF, high frequency power; LFnu, LF in normalized units. (DOCX) [file pone.0177042.s003.docx]

**S3 Table.** A**ssociations for occupational (OPA) and leisure-time physical activity (LTPA) with heart rate and heart rate variability indices during sleep,** **stratified by Social support at work.**

|  | **Low Support** | | | **High Support** | | |
| --- | --- | --- | --- | --- | --- | --- |
|  | **B** | **SE** | ***p*** | **B** | **SE** | ***p*** |
| **Heart rate (bpm)** |  |  |  |  |  |  |
| **OPA** | 0.60 | 0.69 | 0.382 | 0.44 | 0.85 | 0.608 |
| **LTPA** | -2.24 | 1.18 | 0.061 | -1.11 | 1.50 | 0.461 |
| **Interaction** | 0.67 | 0.15 | 0.000 | 0.40 | 0.18 | 0.026 |
| **RMSSD (ln ms)** |  |  |  |  |  |  |
| **OPA** | -0.02 | 0.05 | 0.664 | 0.01 | 0.06 | 0.895 |
| **LTPA** | 0.10 | 0.09 | 0.269 | 0.05 | 0.11 | 0.666 |
| **Interaction** | -0.02 | 0.01 | 0.037 | -0.03 | 0.01 | 0.033 |
| **SDNN (ms)** |  |  |  |  |  |  |
| **OPA** | -1.43 | 2.20 | 0.516 | 2.97 | 2.61 | 0.258 |
| **LTPA** | 3.37 | 3.79 | 0.375 | 0.95 | 4.59 | 0.836 |
| **Interaction** | -0.73 | 0.49 | 0.134 | -0.96 | 0.55 | 0.083 |
| **LF (ln ms^2^)** |  |  |  |  |  |  |
| **OPA** | -0.13 | 0.09 | 0.180 | 0.00 | 0.10 | 0.999 |
| **LTPA** | 0.17 | 0.16 | 0.289 | 0.28 | 0.18 | 0.122 |
| **Interaction** | -0.03 | 0.02 | 0.120 | -0.03 | 0.02 | 0.110 |
| **HF (ln ms^2^)** |  |  |  |  |  |  |
| **OPA** | -0.10 | 0.11 | 0.35 | 0.03 | 0.13 | 0.81 |
| **LTPA** | 0.18 | 0.18 | 0.33 | -0.01 | 0.23 | 0.98 |
| **Interaction** | -0.03 | 0.02 | 0.21 | -0.05 | 0.03 | 0.08 |
| **LFnu** |  |  |  |  |  |  |
| **OPA** | 0.00 | 0.02 | 0.92 | 0.00 | 0.02 | 0.82 |
| **LTPA** | -0.01 | 0.03 | 0.73 | 0.05 | 0.04 | 0.15 |
| **Interaction** | 0.00 | 0.00 | 0.98 | 0.00 | 0.00 | 0.44 |

Note: Estimates (B) represent change in HRV indices with 10 unit increments in percent time in OPA and LTPA, which were centered prior to the analysis; Interaction represents OPA × LTPA; the models are adjusted for age, gender, body-mass index and current smoking.

Abbreviations: RMSSD, root mean squared successive differences between RR intervals; SDNN, standard deviation of RR intervals; LF, low frequency power, HF, high frequency power; LFnu, LF in normalized units.
