# Supplementary material for: On the health paradox of occupational and leisure-time physical activity using objective measurements: Effects on autonomic imbalance
Source: PLoS One. 2017 May 4;12(5):e0177042. doi: 10.1371/journal.pone.0177042 (PMC5417644; doi:10.1371/journal.pone.0177042)
Supplement: S4 Table — Note: Estimates (B) represent change in HRV indices with 10 unit increments in percent time in OPA and LTPA, which were centered prior to the analysis; Interaction represents OPA × LTPA; the models are adjusted for age, gender, body-mass index and current smoking. Abbreviations: RMSSD, root mean squared successive differences between RR intervals; SDNN, standard deviation of RR intervals; LF, low frequency power, HF, high frequency power; LFnu, LF in normalized units. (DOCX) [file pone.0177042.s004.docx]

**S4 Table.** A**ssociations for occupational (OPA) and leisure-time physical activity (LTPA) with heart rate and heart rate variability indices during sleep,** **stratified by the occurrence of cardiovascular disease (CVD).**

|  | **No CVD 1 (n=345)** | | | **CVD (n=155)** | | |
| --- | --- | --- | --- | --- | --- | --- |
|  | **B** | **SE** | ***p*** | **B** | **SE** | ***p*** |
| **Heart rate (bpm)** |  |  |  |  |  |  |
| **OPA** | 0.70 | 0.49 | 0.159 | 1.61 | 0.99 | 0.105 |
| **LTPA** | -1.28 | 0.84 | 0.129 | -2.25 | 1.73 | 0.197 |
| **Interaction** | 0.35 | 0.11 | 0.001 | 1.04 | 0.26 | 0.000 |
| **RMSSD (ln ms)** |  |  |  |  |  |  |
| **OPA** | -0.01 | 0.04 | 0.834 | -0.12 | 0.07 | 0.080 |
| **LTPA** | 0.05 | 0.06 | 0.482 | 0.04 | 0.12 | 0.744 |
| **Interaction** | -0.02 | 0.01 | 0.011 | -0.04 | 0.02 | 0.054 |
| **SDNN (ms)** |  |  |  |  |  |  |
| **OPA** | 0.40 | 1.65 | 0.808 | -6.43 | 2.54 | 0.013 |
| **LTPA** | 1.62 | 2.80 | 0.563 | -2.33 | 4.47 | 0.602 |
| **Interaction** | -1.01 | 0.36 | 0.006 | -0.44 | 0.68 | 0.520 |
| **LF (ln ms^2^)** |  |  |  |  |  |  |
| **OPA** | -0.06 | 0.07 | 0.396 | -0.29 | 0.11 | 0.010 |
| **LTPA** | 0.23 | 0.11 | 0.042 | -0.05 | 0.20 | 0.788 |
| **Interaction** | -0.04 | 0.01 | 0.011 | -0.03 | 0.03 | 0.390 |
| **HF (ln ms^2^)** |  |  |  |  |  |  |
| **OPA** | -0.02 | 0.08 | 0.816 | -0.27 | 0.14 | 0.054 |
| **LTPA** | 0.08 | 0.14 | 0.575 | 0.03 | 0.24 | 0.912 |
| **Interaction** | -0.03 | 0.02 | 0.072 | -0.07 | 0.04 | 0.044 |
| **LFnu** |  |  |  |  |  |  |
| **OPA** | -0.01 | 0.01 | 0.638 | 0.00 | 0.02 | 0.959 |
| **LTPA** | 0.02 | 0.02 | 0.273 | -0.02 | 0.04 | 0.572 |
| **Interaction** | 0.00 | 0.00 | 0.812 | 0.01 | 0.01 | 0.074 |

Note: Estimates (B) represent change in HRV indices with 10 unit increments in percent time in OPA and LTPA, which were centered prior to the analysis; Interaction represents OPA × LTPA; the models are adjusted for age, gender, body-mass index and current smoking.

Abbreviations: RMSSD, root mean squared successive differences between RR intervals; SDNN, standard deviation of RR intervals; LF, low frequency power, HF, high frequency power; LFnu, LF in normalized units.
