# Supplementary material for: On the health paradox of occupational and leisure-time physical activity using objective measurements: Effects on autonomic imbalance
Source: PLoS One. 2017 May 4;12(5):e0177042. doi: 10.1371/journal.pone.0177042 (PMC5417644; doi:10.1371/journal.pone.0177042)
Supplement: S5 Table — Note: Estimates (B) represent change in HRV indices with 10 unit increments in percent time in OPA and LTPA, which were centered prior to the analysis; Interaction represents OPA × LTPA; the models are adjusted for age, gender, body-mass index and current smoking. Abbreviations: RMSSD, root mean squared successive differences between RR intervals; SDNN, standard deviation of RR intervals; LF, low frequency power, HF, high frequency power; LFnu, LF in normalized units. (DOCX) [file pone.0177042.s005.docx]

**S5 Table.** A**ssociations for occupational (OPA) and leisure-time physical activity (LTPA) with heart rate and heart rate variability indices during sleep,** **stratified by controlled hypertension.**

|  | **Controlled hypertension (n=25)** | | | **Uncontrolled hypertension (n=70)** | | |
| --- | --- | --- | --- | --- | --- | --- |
|  | **B** | **SE** | ***p*** | **B** | **SE** | ***p*** |
| **Heart rate (bpm)** |  |  |  |  |  |  |
| **OPA** | -3.94 | 2.54 | 0.144 | 1.70 | 1.59 | 0.289 |
| **LTPA** | -1.25 | 4.85 | 0.801 | -4.46 | 2.91 | 0.131 |
| **Interaction** | 1.21 | 0.81 | 0.157 | 1.16 | 0.41 | 0.007 |
| **RMSSD (ln ms)** |  |  |  |  |  |  |
| **OPA** | 0.07 | 0.21 | 0.753 | -0.13 | 0.12 | 0.255 |
| **LTPA** | -0.06 | 0.40 | 0.889 | 0.12 | 0.21 | 0.581 |
| **Interaction** | -0.06 | 0.07 | 0.401 | -0.06 | 0.03 | 0.040 |
| **SDNN (ms)** |  |  |  |  |  |  |
| **OPA** | 0.32 | 6.55 | 0.962 | -5.39 | 4.54 | 0.240 |
| **LTPA** | -14.02 | 12.51 | 0.283 | -1.27 | 8.31 | 0.879 |
| **Interaction** | -3.18 | 2.08 | 0.151 | -1.10 | 1.18 | 0.358 |
| **LF (ln ms^2^)** |  |  |  |  |  |  |
| **OPA** | -0.24 | 0.26 | 0.363 | -0.20 | 0.20 | 0.319 |
| **LTPA** | -0.35 | 0.49 | 0.490 | 0.05 | 0.37 | 0.890 |
| **Interaction** | -0.11 | 0.08 | 0.213 | -0.04 | 0.05 | 0.431 |
| **HF (ln ms^2^)** |  |  |  |  |  |  |
| **OPA** | 0.03 | 0.44 | 0.947 | -0.32 | 0.22 | 0.150 |
| **LTPA** | -0.24 | 0.84 | 0.780 | 0.35 | 0.41 | 0.387 |
| **Interaction** | -0.06 | 0.14 | 0.677 | -0.14 | 0.06 | 0.022 |
| **LFnu** |  |  |  |  |  |  |
| **OPA** | -0.06 | 0.07 | 0.403 | 0.03 | 0.03 | 0.294 |
| **LTPA** | -0.02 | 0.13 | 0.857 | -0.06 | 0.06 | 0.355 |
| **Interaction** | -0.01 | 0.02 | 0.593 | 0.02 | 0.01 | 0.019 |

Note: Estimates (B) represent change in HRV indices with 10 unit increments in percent time in OPA and LTPA, which were centered prior to the analysis; Interaction represents OPA × LTPA; the models are adjusted for age, gender, body-mass index and current smoking.

Abbreviations: RMSSD, root mean squared successive differences between RR intervals; SDNN, standard deviation of RR intervals; LF, low frequency power, HF, high frequency power; LFnu, LF in normalized units.
